# Supplementary material for: Clinical Management of Pathogen-Negative Tuberculous Meningitis in Adults: A Series Case Study
Source: J Clin Med. 2022 Oct 23;11(21):6250. doi: 10.3390/jcm11216250 (PMC9656908; doi:10.3390/jcm11216250)
Supplement: Supplementary file 1 [file jcm-11-06250-s001.zip › jcm-1932503-supplementary.pdf]

Supplement Table S1. Details of lumbar puncture time and various index values in CSF for five patients.

	**WCC (10^6/L)**	**N %**	**L %**	**M %**	**Glu (mmol/ L×10^{-2})**	**Blood Glu (mmol/ L×10^{-2})**	**CSF/ Blood glucose**	**Lactate (mmol/ L×10^{-2})**	**LDH (U/L)**	**Protein (mg/L)**	**Cl^- (mmol/L)**	**IgG (mg/L)**	**IgM (mg/L)**	**IgA (mg/L)**
**Case1**														
05/06	350	5	90	5	138	650	0.21	472	36	1383	107.5	122	5.2	43.1
05/14	440	0	95	5	189	580	0.33	412	33	1265	109.8	/	/	/
05/25	780	8	87	5	139	540	0.26	366	24	1232	107	151	4.3	35.8
06/03	400	22	74	4	169	470	0.36	402	24	960	109.6	131	4.6	31.5
06/15	190	9	87	4	205	680	0.30	360	21	1082	113.4	116	4.5	31.3
06/28	41	-	-	-	225	760	0.30	271	19	918	121.6	76.9	2.7	25.8
08/06	20	-	-	-	286	940	0.30	172	19	859	123.8	71.5	1.8	21.6
09/22	65	0	94	6	289	860	0.34	157	18	768	127.3	63	1.2	13
**Case2**														
04/23	510	15	84	1	208	700	0.30	583	47	1683	120	162	5	13.4
04/30	330	12	85	3	216	700	0.31	332	36	1084	118.3	104	5	10.3
05/07	550	10	88	2	261	920	0.28	393	44	1399	122	205	8.7	17.9
05/14	160	6	91	3	243	820	0.30	373	22	1252	121.5	178	5.4	15.7
05/21	230	2	98	0	283	1010	0.28	339	20	1272	120.3	160	4.3	14
05/28	460	11	89	0	242	1040	0.12	324	18	1220	120.3	181	5.8	16
06/04	48	-	-	-	232	930	0.25	290	18	1107	119.4	156	3.6	11.8
06/11	65	2	96	2	261	660	0.40	270	19	1204	122.8	163	3	11

06/30	110	6	89	5	261	780	0.33	221	20	1043	123.1	125	2.2	9.3
07/30	49	-	-	-	338	740	0.46	214	20	859	126.3	104	1.1	7.7
**Case3**														
04/18	134	10	90	0	196	890	0.22	452	49	2356	112.1	178	14.4	20.7
04/25	160	8	88	4	202	630	0.32	359	57.00	980	113.1	69	5.9	6.8
05/02	60	6	90	4	239	860	0.28	338	25	579	116.4	45.1	3.1	5.1
05/27	0	-	-	-	340	1110	0.31	250	26	388	120.6	27.9	0.8	2.4
10/22	0	-	-	-	347	850	0.41	189	24	317	127.6	19.1	0.2	0.7
**Case4**														
08/03	250	40	58	2	151	750	0.20	535	113.3	1260	113.3	118	4.4	24.7
08/10	45	-	-	-	216	770	0.28	370	17	919	120	81	5.2	17.4
08/31	20	-	-	-	234	830	0.28	332	21	1277	116.6	143	8.9	27.2
10/13	25	-	-	-	283	970	0.29	299	29	911	116.7	90.2	7.6	16.8
02/08	4	-	-	-	337	730	0.46	155	10	356	120.3	35	0.8	6.1
**Case5**														
07/21	125	38	56	6	215	830	0.26	352	39	819	114.4	106	10.4	23.8
07/28	160	21	79		175	910	0.19	280	50	900	116.9	/	/	/
08/04	300	18	75	6	160	1290	0.12	317	52	823	117.1	/	/	/
08/13	120	6	94		272	1430	0.19	270	38	373	117.1	/	/	/
08/23	44	-	-	-	283	720	0.39	223	31	353	117.6	49.1	＞60	10.6
10/08	1	-	-	-	317	540	0.59	174	30	266	123.5	21.7	8.7	3.4

Legend: WCC—white cells count in the CSF (10^6/L);N%—the percentage of Neutrophils; L%—the percentage of lymphocyte; M%—the percentage of monocyte; Glu—Glucose concentration in the CSF(mmol/L×10^{-2}); Lactate—Lactate concentration in the CSF(mmol/L×10-2); LDH —Lactate dehydrogenase concentration in the CSF(U/L); Protein—Protein concentration in the CSF (mg/L); Cl^-—chlorine ions concentration in the CSF (mmol/L); IgG/IgM/IgA—Immunoglobulin G/ Immunoglobulin M/ Immunoglobulin A concentration in the CSF(mg/L); "-"— When the total number of white cells is less than 50 x10^6/L, the cells are not classified; "/"— not performed in this patient.

Referential values: WCC: 0-8×10^6/L; Glucose: 222-389 mmol/L×10^{-2}; Lactate:110-240 mmol/L×10^{-2}; Lactate dehydrogenase: ＜40 U/L; Protein: 150–450 mg/L; Cl^{-}:120–130 mmol/L; IgG ≤58.6 mg/L; IgM ≤7 mg/L; IgA ≤7 mg/L;

Supplement Table S2. The Lancet consensus scoring system for the five patients

	**Diagnostic score**	**Case1**	**Case2**	**Case3**	**Case4**	**Case5**
Clinical criteria	(Maximum category score=6）	case1	case2	case3	case4	case5
Symptom duration of more than 5 days	4	4	4	4	4	4
Systemic symptoms suggestive of tuberculosis (one or more of the following): weight loss (or poor weight gain in children), night sweats, or persistent cough for more than 2 weeks	2	2	0	0	0	0
History of recent (within past year) close contact with an individual with pulmonary tuberculosis or a positive TST or IGRA (only in children <10 years of age)	2	0	0	0	0	0
Focal neurological deficit (excluding cranial palsies)	1	0	0	0	0	1
Cranial nerve palsy	1	1	0	0	0	1
Altered consciousness	1	1	1	1	0	1
CSF criteria	(Maximum category score=4）					
Clear appearance	1	1	1	1	1	1
Cells: 10－500 per μl	1	1	1	1	1	1
Lymphocytic predominance (>50%)	1	1	1	1	1	1
Protein concentration greater than 1 g/L	1	1	1	1	1	0
CSF to plasma glucose ratio of less than 50% or an absolute CSF glucose concentration less than 2.2 mmol/L	1	1	1	1	1	0
Cerebral imaging criteria	(Maximum category score=6）					
Hydrocephalus	1	0	0	0	0	0
Basal meningeal enhancement	2	2	0	0	0	0
Tuberculoma	2	2	2	0	2	0
Infarct	1	0	0	0	0	0
Pre-contrast basal hyperdensity	2	0	0	0	0	0

Evidence of tuberculosis elsewhere	(Maximum category score=4）					
Chest radiograph suggestive of active tuberculosis signs of tuberculosis = 2; miliary tuberculosis = 4	4	0	0	0	0	0
CT/MRI/Ultrasound evidence for tuberculosis outside the CNS	2	0	0	0	0	0
AFB identified or Mycobacterium tuberculosis cultured from another source-i.e., sputum, lymph node, gastric washing, urine, blood culture	4	0	0	0	0	0
Positive commercial M. tuberculosis NAAT from extra-neural specimen	4	0	0	0	0	0
Exclusion of alternative diagnoses*						
**Total score**		**17**	**12**	**10**	**11**	**10**

“*”—An alternative diagnosis must be confirmed microbiologically (by stain, culture, or NAAT when appropriate), serologically(eg, syphilis), or histopathologically (eg, lymphoma). The list of alternative diagnoses that should be considered, dependent upon age, immune status, and geographical region, include: pyogenic bacterial meningitis, cryptococcal meningitis, syphilitic meningitis, viral meningo-encephalitis, cerebral malaria, parasitic or eosinophilic meningitis (Angiostrongylus cantonesis, Gnathostoma spinigerum, toxocariasis, cysticercosis), cerebral toxoplasmosis and bacterial brain abscess (space-occupying lesionon cerebral imaging)and malignancy (eg, lymphoma)

Supplement Table S3. The Thwaites'system for the five patients

	Diagnostic index	Case1	Case2	Case3	Case4	Case5
Age (years)						
≥36	2	2	2	2	2	2
<36	0					
Blood WCC (10^3/mL)						
≥15 000	4					
<15 000		0	0	0	0	0
History of illness (days)						
≥6	−5	−5	−5	−5	−5	-5
<6						
CSF total WCC (10^3/mL)						
≥900						
<900	0	0	0	0	0	0
CSF % neutrophils						
≥75	4					
<75	0	0	0	0	0	0
yes	≤4	-3	-3	-3	-3	-3
no	＞4					

Supplement Table S4. Sixteen cases of pathogen-negative Tuberculosis meningitis reported in the literature from 2020 to date

Tuberculosis cases	Age(year)	Sex	Illness history*	Symptom duration (days)*	Clinical syndrome	CSF results							Evidence of extracranial tuberculosis	Brain-MRI	Treatment	Outcome
						WCC (10^6/L)	Protein (mg/dl)	Glu (mmol/L)	*M.tb* culture	ZN smear	PCR	Xpert				
**Flynn, W. P. et al.* [1]**	30	Male	No	＞30	Weight loss; Headache; Fever; Confused; Lost the ability to speak English	18000	672	0.8	+	-	-	/	No evidence find in Chest-CT/ Abdomen-CT/ Pelvis-CT	Clival osteitis; Communicating Hydrocephalu	0.3g Isoniazid; 0.6g Rifampin; 1g Levofloxacin; 2g Pyrazinamide; Dexamethasone; Linezolid	Complete improvement
**Vasconcelos, G. et al. [2]**	47	Female	No	＞60	Vomiting episode; Asthenia; Weight loss; Disorientation; Psychomotor agitation	120	128	2	-	-	-	/	Miliary tuberculosis	Numerous contrast-enhanced lesions; Leptomeningeal enhancement	Isoniazid; Rifampin; Pyrazinamide; Ethambutol; Dexamethasone	Complete improvement
**Tala-Ighil, T. et al. .[3]**	48	Male	Hypertension;Diabetes	5	Weight loss; Central facial paralysis; Dysarthria; Left hemiplegia	5	105	/	-	-	-	/	Miliary tuberculosis	Acute infarction of internal capsule	Isoniazid; Rifampin; Pyrazinamide; Ethambutol; Prednisone	Partial recovery
**Esposito, S. B. et al. [4]**	30	Male	No	＞14	Weight loss; Vomiting; Headache; Fever	/	351	0.7	-	-	-	/	Miliary tuberculosis; Multiple ring-enhancing foci in spine;sacral osteomyelitis;septic arthritis;	Multiple ring-enhancing lesions	Isoniazid; Rifampicin; Pyrazinamide; Ethambutol; Pyridoxine; Dexamethasone	/

												paraspinous abscess			
**Chesdachai, S. et al.* [5]**	34	Female	Hypertension;Diabetes	14	Headache; Fever; Diplopia; Blurred vision; Confused	955	179	1.8	+	-	+	-	No evidence find in Chest x-ray	Leptomeningeal enhancement; Acute cerebral infarction; Communicating hydrocephalu	Isoniazid; Rifampin; Pyrazinamide; Ethambutol	Partial recovery
**Shao, Kai. et al. [6]**	46	Male	Hypertension	＞30	Headache; Fever; Anorexia; Asthenia; One-and-a-half syndrome; Right peripheral facial nerve palsy; Horner’s syndrome	756	81.92	2.81	-	-	-	/	No evidence find in Chest-CT	Numerous contrast-enhancement in the medulla oblongata, pons, and leptomeninge	0.4g Isoniazid 0.45g Rifampin 10mg Dexamethasone	Complete improvement
**Zafar, Z. et al. [7]**	24	Male	No	＞30	Headache; Fever; Confused; A solitary seizure; Paralysis of both lower limbs; Dysuria	/	298	1.16	-	-	-	-	Enhanced nodules and ring lesions extending from thoracic segment T2 to T9.	Basal meningeal enhancement	0.75g Isoniazid； 0.15g Rifampicin； 0.4g Pyrazinamide； 0.275g Ethambutol； 10mg Dexamethasone	Partial recovery
**Kitazaki, Yuki. et al. [8]**	70	Female	No	＜10	Cough; Fever; Impaired consciousness; Right-sided hemiparesis, Conjugate eye deviation to the right	760	273	0.72	-	-	-	-	/	Diffuse thickness of the leptomeninges; Nodular lesions with gadolinium enhancement around the brainstem	0.3g Isoniazid； 0.45g Rifampicin； 1.2g Pyrazina-mide； 0.75g Ethambutol； 32mg Dexamethasone	Died

**Oka, Y. et al. [9]**	61	Female	chronic renal failure	7	Fatigue; Fever; Deteriorated; Nonconvulsive Status Epilepticus	225	405.7	0.56	-	-	+	-	No evidence find in Chest-CT	Transient diffusion restriction	Isoniazid; Rifampicin; Ethambutol	Partial recovery
**Elavarasi, A. et al. [10]**	26	Female	No	＞60	Headache; Fever	540	260	0.83	-	-	-	-	No evidence find in Chest-X-ray	Multiple enhancing lesions in brain stem	Isoniazid; Rifampicin; Pyrazinamide; Ethambutol	Complete improvement
**Gaba, S. et al.[11]**	20	Female	No	14	Anorexia; Headache; Fever	194	160	2.44	-	-	+	-	No evidence find in Chest-X-ray	Focal temporal cerebritis with meningitis; Cerebellar granulomas; Basal exudates	Isoniazid; Rifampicin; Pyrazinamide; Ethambutol	Partial recovery
**Siahaan, A. M. P. et al.[12]**	38	Male	No	14	Headache; Fever; Cough; Weight loss; Right Hemiparesis; Paroxysmal sympathetic hyperactivity; Loss of consciousness	/	320	2.89	-	-	-	/	Pulmonary tuberculosis	Basal cistern enhancement; Rim enhancement mass on the quadrigeminal cistern; Communicating hydrocephalus	Rifampicin; Isoniazid; Pyrazinamide; Ethambutol; Streptomycin	Partial recovery
**Desai, N. et al. [13]**	53	Female	vulvar squamous cell carcinoma, hypertension, type 2 diabetes	21	Headache; Fatigue; Diplopia; Slurred speech; Mild confusion	72	142	5.5	-	-	-	/	No evidence find in Chest-CT	Multiple ring-enhancing lesions	Rifampin; Isoniazid; Pyrazinamide; Ethambutol; Dexamethasone	Complete improvement
**Bongomin, F. et al. [14]**	20	Male	No	14	Headache; Fever; Bilateral sixth cranial nerve palsy; Urinary retention; Horner's syndrome	80	/	6.4	-	-	/	+	No evidence find in Chest-CT	Diffuse sulcal effacement with predominant basal contrast meningeal enhancement	Rifampicin; Isoniazid; Pyrazinamide; Ethambutol; Pyridoxine	Complete improvement
**Arif, S. et al. [15]**	65	Male	Hypertension	60	Anorexia; Weight loss; Headache;	250	135	1.78	-	-	+	/	No evidence find in Chest-CT/ Abdomen-CT/ Pelvis-CT	Extensive ring enhancement lesions	Isoniazid; Rifampicin; Ethambutol; Pyrazinamide;	Complete improvement

					Fever;<br>Disorientation										Dexamethasone;<br>Moxifloxacin	
**Kaur, H. et al. [16]**	28	Female	No	36	Weight loss;<br>Drowsy;<br>Headache;<br>Fever	120	1332.8	2.16	-	-	/	-	No evidence find in Chest-CT	Leptomeningeal enhancement;<br>Conglomerate ring enhancing lesions in the extramedullary intradural compartment	Isoniazid;<br>Rifampicin;<br>Pyrazinamide;<br>Ethambutol;<br>Streptomycin;<br>Prednisolone	Partial recovery

Legend: Illness history*—diabetes, hepatitis B, AIDS, syphilis, Immune-mediated rheumatic disease, organ transplantation; Syndrome duration*—the time from symptoms onset to admission;
Flynn, W. P. et al.*—*M.tb* was isolated in the CSF taken from lumbar puncture on admission after 35 days of incubation;
Chesdachai, S. et al.*— On hospital day 17, The patient was empirically started. On hospital day 24, *M.tb* PCR positive. On hospital day 27, *M.tb* culture from the second LP (hospital day 9) positive;

References

1. Flynn, W. P.; Ntuli, Y.; Zhang, H.; Tiberi, S. A case of Clival Tuberculosis and associated meningitis. *J Clin Tuberc Other Mycobact Dis* **2021,** *25*, 100273.
2. Vasconcelos, G.; Santos, L.; Couto, C.; Cruz, M.; Castro, A. Miliary Brain Tuberculomas and Meningitis: Tuberculosis Beyond the Lungs. *Eur J Case Rep Intern Med* **2020,** *7*, 001931.
3. Tala-Ighil, T.; Greffe, S.; Trad, S.; Delaroche, M.; Coutte, L.; Rouveix, E.; Kahn, J. E.; Hanslik, T. [Cerebral infarction and tuberculosis: case report and literature review]. *Rev Med Interne* **2020,** *41*, 704-707.
4. Esposito, S. B.; Levi, J.; Matuzsan, Z. M.; Amaducci, A. M.; Richardson, D. M. A Case Report of Widely Disseminated Tuberculosis in Immunocompetent Adult Male. *Clin Pract Cases Emerg Med* **2020,** *4*, 375-379.
5. Chesdachai, S.; Katz, B.; Sapkota, S. Diagnostic Challenges and Dilemmas in Tuberculous Meningitis. **2020,** *359*, 6.
6. Shao, K.; Dong, F.; Guo, S.; Wang, J.; Sun, Z. Eight-and-a-half syndrome caused by tuberculous meningitis: a case report. *Acta Neurologica Belgica* **2021,** *121*, 591-593.
7. Zafar, Z.; Hafeez, M. A.-O.; Butt, M. Elusive tuberculous meningitis with rare neurological complication of longitudinally extensive transverse myelitis: a case report. *Spinal Cord Ser. cases* **2021,** *14*, 82.
8. Kitazaki, Y.; Ikawa, M.; Enomoto, S.; Shirafuji, N.; Hayashi, K.; Yamamura, O.; Yamada, S.; Arishima, H.; Noriki, S.; Nakamoto, Y.; et al. An autopsy case of tuberculous meningitis undiagnosed by nested-PCR of CSF samples and brain biopsy. *Journal of the Neurological Sciences* **2020,** *415*, 116968.
9. Oka, Y.; Tabu, H.; Matsumoto, S. Tuberculous meningitis presenting with nonconvulsive status epilepticus and transient diffusion restriction: A rare case. *Neurol India.* **2020,** *68*, 512.
10. Elavarasi, A.; Goyal, V. Brainstem tuberculoma: A delayed IRIS. *Indian J Tuberc* **2020,** *67*, 343-345.
11. Gaba, S.; Gupta, M.; Lamba, A. S.; Bhardwaj, A.; Gupta, H. Bilateral Complete Oculomotor Palsy in Tubercular Meningitis. *Cureus.* **2020,** *12*, 11001.
12. Siahaan, A. M. P.; Tandean, S.; Indharty, R. S.; Nainggolan, B. W. M.; Susanto, M. Paroxysmal sympathetic hyperactivity syndrome in tuberculous meningitis with paradoxical reaction. *Int J Surg Case Rep* **2022,** *99*, 107619.
13. Desai, N.; Krishnan, R.; Rukmangadachar, L. Central Nervous System Tuberculosis Presenting With Multiple Ring-Enhancing Lesions: A Diagnostic Challenge. *Cureus.* **2022,** *14*, 21819.
14. Bongomin, F.; Khan, S. A.; Oravec, T. A Complete Triad: Horner's Syndrome in Tuberculous Meningitis. *Am J Med Sci* **2020,** *360*, 204-205.
15. Arif, S.; Arif, S.; Slehria, A. U.; Yousaf, G.; Nawaz, K. H., Sr. Central Nervous System Tuberculosis With Shower Like Pattern of Intracranial Tuberculomas in an Immunocompetent Patient. *Cureus.* **2020,** *12*, 9922.
16. Kaur, H.; Mittal, G. K.; Singhdev, J. Intradural extramedullary tuberculoma of the spinal cord in patient of tubercular meningitis - an uncommon scenario. *Indian J Tuberc* **2020,** *67*, 426-429.
